# Selective increases of AMPA, NMDA, and kainate receptor subunit mRNAs in the hippocampus and orbitofrontal cortex but not in prefrontal cortex of human alcoholics

**DOI:** 10.3389/fncel.2014.00011

**Published:** 2014-01-29

**Authors:** Zhe Jin, Amol K. Bhandage, Igor Bazov, Olga Kononenko, Georgy Bakalkin, Esa R. Korpi, Bryndis Birnir

**Affiliations:** ^1^Department of Neuroscience, Uppsala UniversityUppsala, Sweden; ^2^Division of Biological Research on Drug Dependence, Department of Pharmaceutical Biosciences, Uppsala UniversityUppsala, Sweden; ^3^Institute of Biomedicine, Pharmacology, University of HelsinkiHelsinki, Finland

**Keywords:** glutamate receptor, ethanol, GluR, glutamate, excitatory, ion channel

## Abstract

Glutamate is the main excitatory transmitter in the human brain. Drugs that affect the glutamatergic signaling will alter neuronal excitability. Ethanol inhibits glutamate receptors. We examined the expression level of glutamate receptor subunit mRNAs in human post-mortem samples from alcoholics and compared the results to brain samples from control subjects. RNA from hippocampal dentate gyrus (HP-DG), orbitofrontal cortex (OFC), and dorso-lateral prefrontal cortex (DL-PFC) samples from 21 controls and 19 individuals with chronic alcohol dependence were included in the study. Total RNA was assayed using quantitative RT-PCR. Out of the 16 glutamate receptor subunits, mRNAs encoding two AMPA [2-amino-3-(3-hydroxy-5-methyl-isoxazol-4-yl)propanoic acid] receptor subunits GluA2 and GluA3; three kainate receptor subunits GluK2, GluK3 and GluK5 and five NMDA (N-methyl-D-aspartate) receptor subunits GluN1, GluN2A, GluN2C, GluN2D, and GluN3A were significantly increased in the HP-DG region in alcoholics. In the OFC, mRNA encoding the NMDA receptor subunit GluN3A was increased, whereas in the DL-PFC, no differences in mRNA levels were observed. Our laboratory has previously shown that the expression of genes encoding inhibitory GABA-A receptors is altered in the HP-DG and OFC of alcoholics (Jin et al., 2011). Whether the changes in one neurotransmitter system drives changes in the other or if they change independently is currently not known. The results demonstrate that excessive long-term alcohol consumption is associated with altered expression of genes encoding glutamate receptors in a brain region-specific manner. It is an intriguing possibility that genetic predisposition to alcoholism may contribute to these gene expression changes.

## Introduction

Alcohol is probably the most commonly consumed addictive substance today and is often abused. Long-term excessive consumption of alcohol modifies the brain and may lead to behavioral changes, addiction and cognitive dysfunction (Harper, 1998). Magnetic resonance imaging studies have shown reduced hippocampal and prefrontal cortex volume in individuals with alcohol dependence. These changes in brain structures may contribute to the cognitive deficit associated with chronic alcohol exposure and may be related to effects of alcohol on several neurotransmitter systems (Jernigan et al., 1991; Sullivan et al., 1995; Harris et al., 2008; Vengeliene et al., 2008; Spanagel, 2009). Alcohol alters excitatory signaling at glutamate-activated ionotropic glutamate receptors (Smart and Paoletti, 2012) but despite overwhelming evidence that glutamatergic transmission is involved in alcoholism (Gass and Olive, 2008), it is still not clear how synaptic transmission is altered by chronic alcohol consumption and how the effects may vary between different brain regions.

Glutamate receptors are subgrouped into AMPA, kainate, NMDA, and delta families of receptors. The receptors form ion channels that are gated by glutamate. The AMPA receptors normally mediate fast synaptic transmission and synaptic strength whereas the NMDA receptors once relieved of the Mg^2+^ block have high calcium permeability and, thereby, regulate intracellular signaling, and synaptic plasticity. The kainate receptors are present both in the pre- and post-synaptic terminals and are thought to have a modulatory role and presynaptically influence transmitter release (Moykkynen and Korpi, 2012; Smart and Paoletti, 2012). The delta receptors are electrically silent and to-date have no clear function in neurons (Moykkynen and Korpi, 2012; Smart and Paoletti, 2012).

Ethanol inhibits the ion channel function of glutamate receptors by both decreasing the peak-current amplitude and accelerating the rate of the current desensitization and, thereby, inhibiting several forms of neuronal plasticity (Lovinger et al., 1989; Gass and Olive, 2008; Moykkynen and Korpi, 2012). There are four AMPA (GluA1-4), five kainate (GluK1-5), seven NMDA (GluN1, GluN2A-D, GluN3A-B), and two delta (GluD1, GluD2) receptors genes. The glutamate receptors are formed by four subunits normally originating from the same family. As the subunits can co-assemble in various constellations, a large number of glutamate receptor subtypes can potentially be formed (Smart and Paoletti, 2012). What glutamate receptor subtypes are active in a neuron depends on which genes are transcribed and this can vary according to developmental stage, brain region, type of neuron, synaptic activity, and even disease as alcoholism (Gass and Olive, 2008; Moykkynen and Korpi, 2012).

Lovinger et al. (1989) demonstrated that the ion channel function of NMDA receptors was inhibited by alcohol in a concentration dependent manner over a range of concentrations that also produces intoxication. A number of other studies have since confirmed and further identified the NMDA receptors as the primary molecular targets of alcohol (Gass and Olive, 2008), but even AMPA and kainate receptors are affected by high consumption of alcohol. Ethanol inhibits several forms of neuronal plasticity including long-term potentialtion (LTP) in the hippocampus which requires activation of both AMPA and NMDA-type glutamate receptors (Blitzer et al., 1990; Morrisett and Swartzwelder, 1993; Givens and McMahon, 1995; Gass and Olive, 2008). Alcohol-dependent disorders have been extensively studied in animal models (see e.g., Vengeliene et al., 2008 and references therein), but the human alcoholism is not fully mimicked by these animal models (Crabbe et al., 2013). Candidate glutamate receptor subunits that have, so far, been identified in human studies of alcohol-related disorders include GluN1, GluN2A, GluN2C, and GluK3 (Mayfield et al., 2002; Flatscher-Bader et al., 2005, 2006; Kalsi et al., 2009; Domart et al., 2012). These studies, conducted on samples from post-mortem brains of alcoholics, are very valuable as they aid in the understanding of the mechanisms underlying human alcohol dependence.

In the present study, we performed reverse transcription quantitative PCR (RT-qPCR) to investigate the expression of glutamate receptor mRNAs in post-mortem tissue. Brain samples from hippocampal dentate gyrus, orbitofrontal cortex and dorsolateral prefrontal cortex of alcoholics were compared with brain samples from individuals without alcohol addiction. The results show that in brain tissue from alcoholics, the levels of glutamate receptor subunit mRNAs were altered in specific areas as compared with controls. In the dentate gyrus region of the hippocampus (HP-DG) mRNA levels of 10 different glutamate receptor subunits were increased whereas only the mRNA encoding GluN3A was increased in the orbitofrontal cortex (OFC) and none in the prefrontal cortex (PFC).

## Methods

### Human postmortem samples

Twenty one human controls and 19 individuals with chronic alcohol dependence were included in the study. All individuals were Caucasian males. The individuals with alcoholism had consumed ≥80 g alcohol per day during the majority of their adult lives, met the criteria for Diagnostic and Statistical Manual for Mental Disorders, 4th edition and National Health and Medical Research Council/World Health Organization and did not have liver cirrhosis, Wernicke-Korsakoff's syndrome, or multi-drug abuse history. Individuals in the control group had either abstained from alcohol completely or were social drinkers who had consumed ≤20 g of alcohol per day on average. Individuals in the control group were matched to individuals with alcoholism by age and post-mortem interval (PMI). Post-mortem brain samples (300–500 mg) from hippocampal dentate gyrus (including both granule and molecular layer, HP-DG), orbitofrontal cortex (Brodmann's area 47, OFC), and dorso-lateral prefrontal cortex (Brodmann's area 9, DL-PFC) were collected at the New South Wales Tissue Resource Center (TRC), University of Sydney, Australia (Sheedy et al., 2008) (http://sydney.edu.au/medicine/pathology/trc/index.php). Samples from all three brain regions were collected from the same donors in 7 controls and 10 alcoholics. All sample areas were identified and then collected by qualified pathologists under full ethical clearance and with informed, written consent from the next of kin. Samples were frozen and kept at −80°C. The detailed demographic data for all subjects are given in supplementary Table S1. The samples are the same, both for controls and the alcoholics, as were used in a study to analyze effects of alcoholism on expression of GABA-A receptor subunit genes (Jin et al., 2011).

### Total RNA isolation

Total RNA was isolated by using RNeasy Lipid Tissue Mini Kit (QIAGEN, Maryland, USA) or GenElute total RNA Miniprep (Sigma) and quantified with Nanodrop (Nanodrop Technlogies, Inc). The quality of RNA was evaluated by measuring RNA Quality Indicator (RQI) using Bio-Rad Experion (Bio-Rad Laroratories, Hercules, CA) with Eukaryote Total RNA StdSens assay following the manufacturer's manual. RQI is equivalent to RNA integrity number (RIN) from Agilent (Denisov et al., 2008). RNA samples with RQI values greater than 5 are suitable for RT-qPCR (Fleige and Pfaffl, 2006). In this study, samples with RQI less than 5 were not used for experiments. Average RQI of all samples was 7.29 ± 0.12 (mean ± SEM) (83% of the samples had RQI greater than 6) indicating high quality of isolated total RNA.

### Quantitative real-time RT-PCR

Total RNA (250 ng) was reverse transcribed into cDNA in a 20 μ l reaction mixture using Superscript III reverse transcriptase (Invitrogen). Negative control was performed by omitting reverse transcriptase in the reaction in order to confirm no genomic DNA contamination in the isolated RNA. qPCRs were done in a 10 μ l reaction mixture containing 4 μ l cDNA (1 ng), 1 × PCR reaction buffer, 3 mM MgCl_2_, 0.3 mM dNTP, 1 × ROX reference dye, 0.8 U JumpStart *Taq* DNA polymerase (Sigma-Aldrich), 5 × SYBR Green I (Invitrogen) and 0.4 μ M each of forward and reverse primers. The gene-specific primer pairs (primer sequences shown in supplementary Table S2) were designed using NCBI Primer-blast and GETPrime (updepla1srv1.epfl.ch/getprime/) synthesized by SigmaAldrich and further validated with cDNA from human brain. Amplification was performed in 384-well optical plates using the ABI PRISM 7900HT Sequence Detection System (Applied Biosystems) with an initial denaturation of 5 min at 95°C, followed by 45 cycles of 95°C for 15 s, 60°C for 30 s, and 72°C for 30 s. A melting curve was determined at the end of cycling to ensure the amplification of a single PCR product. Cycle threshold values (Ct) were determined with the SDS 2.3 and RQ Manager 1.2 softwares supplied with the instrument. The expression of each target gene relative to a normalization factor (geometric mean of two reference genes) was calculated with DataAssist v2.0 using the 2^−ΔCt^ method as previously described (Schmittgen and Livak, 2008). As the expression of reference genes may vary between different brain regions of human alcoholics, it is of great importance to use validated stable reference genes for normalization. The reference genes with an average expression stability value *M* < 0.5 are regarded a stably expressed (Hellemans et al., 2007). Reference genes beta actin (*ACTB*) and ubiquitin C (*UBC*) for HP-DG (average *M* = 0.25), *ACTB* and ribosomal large P0 (*RPLP0*) for DL-PFC (average *M* = 0.22) and phosphoglycerate kinase 1 (*PGK1*) and peptidylprolyl isomerase A (*PPIA*) for OFC (average *M* = 0.125) were selected for normalization according to previously developed approach for analysis of reference genes (Johansson et al., 2007; Kuzmin et al., 2009; Bazov et al., 2011).

### Statistical analysis

Statistical analysis was carried out using Statistica 11 and data were plotted with GraphPad Prism 6. Data were presented in scatter plots with 95% confidence interval or as box and wiskar plots using the Tukey method to determine outliers (data points above or below the whiskers). Statistical analysis was then performed where the outliers were not included when data were compared. Normality of data distribution was analyzed using Shapiro-Wilk normality test (see supplementary Table S3). The differences between groups were assessed by One-Way ANOVA with Bonferroni *post-hoc* test (normally distributed data) or non-parametric Kruskal-Wallis ANOVA on ranks with Dunn's *post-hoc* test (not normally distributed data). A general stepwise linear regression model was used to identify covariates (e.g., age and PMI). Variables with a significant association with group (controls and alcoholics) were included in the final statistical model as covariates. The significance level was set at *p* < 0.05. The sample size was between 11 and 15 that falls within the group-size range providing reliable statistical estimation (Hynd et al., 2003).

## Results

The demographic characteristics of individuals in this study are shown in supplementary Table S1. There was no significant difference in age, PMI, brain pH, RQI, and proportions of smokers and non-smokers between individuals with or without alcohol dependence (supplementary Table S4).

Expression of 16 ionotropic glutamate receptor subunit mRNAs (AMPA subunits: GluA1-4; kainate subunits: GluK1-5; NMDA subunits: GluN1, 2A, 2B, 2C, 2D, 3A, and 3B) was quantified by RT-qPCR in the samples collected from the HP-DG, OFC, and DL-PFC.

### Increased expression of two AMPA, three kainate, and five NMDA receptor subunits mrnas in the hippocampus dentate gyrus region of alcoholics

**Figure 1:**
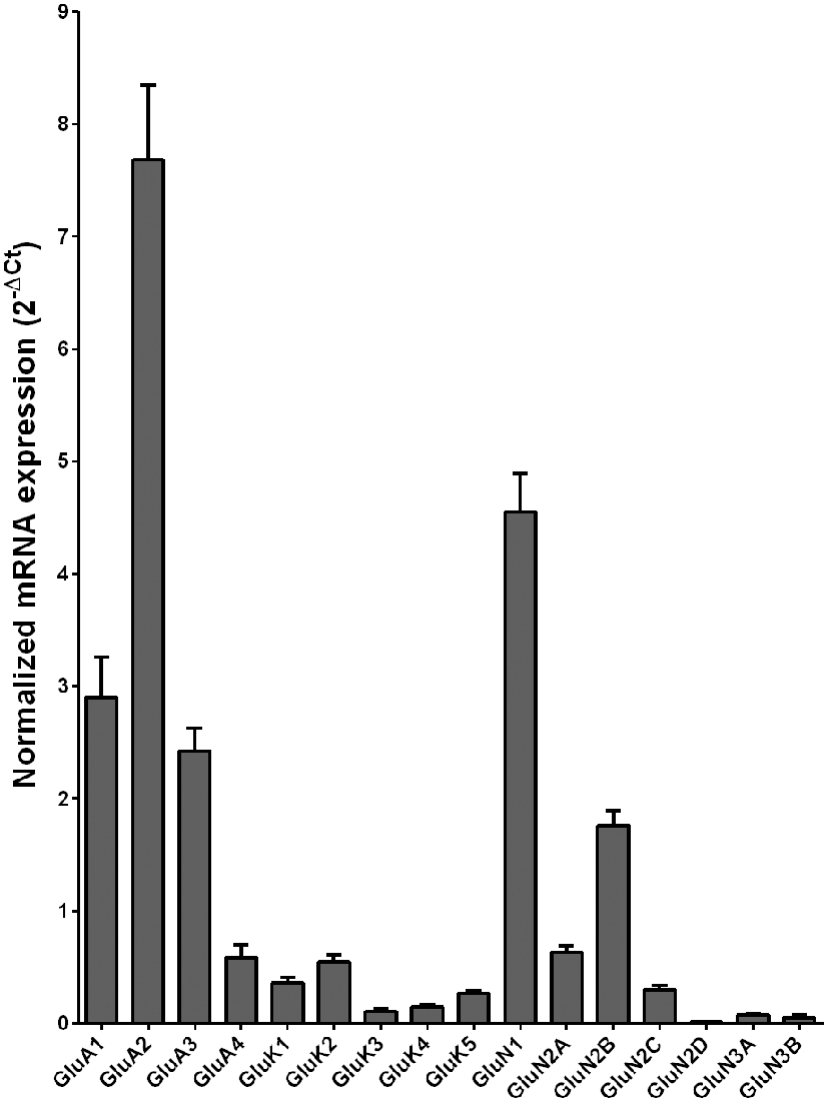
**Expression of ionotropic glutamate receptor subunit mRNAs in the hippocampal dentate gyrus region from control subjects (*n* = 15)**. The mRNA level of each subunit was normalized to reference genes *ACTB* and *UBC* and presented as mean ± SEM.

**Figure 2:**
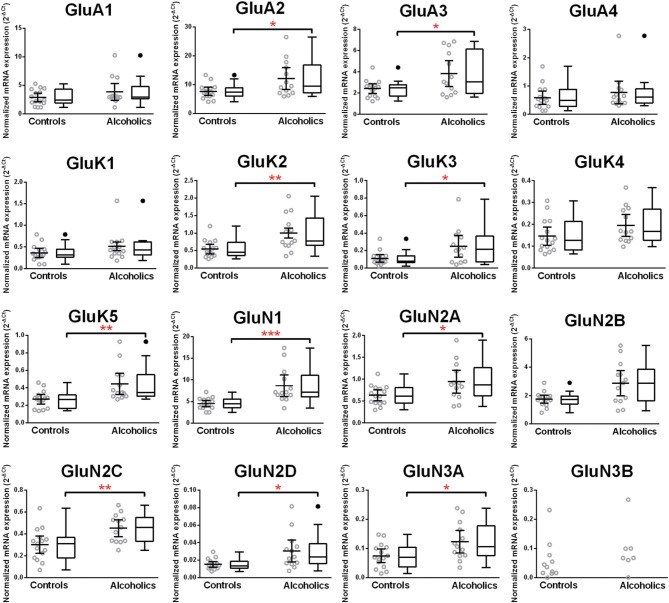
**Expression of ionotropic glutamate receptor subunits mRNA in the hippocampal dentate gyrus of controls (*n* = 15) and alcoholics (*n* = 13)**. Data from each group were presented as scatter dot plot (°) with mean and 95% confidence interval and box and whiskers plot with median and whiskers plotted by Tukey method to determine outliers (•-above or below the whiskers). Statistical analysis was performed after excluding outliers. One Way ANOVA with Bonferroni *post-hoc* test, GluK1, *df* = 24, *p* = 0.11; GluN2C, *df* = 24, *p* = 0.0084; GluN3A, *df* = 24, *p* = 0.012. Kruskal–Wallis ANOVA on ranks with Dunn's *post-hoc* test, GluA1, *H*_(1, 27)_ = 0.40, *p* = 0.53; GluA2, *H*_(1, 27)_ = 5.09, *p* = 0.024; GluA3, *H*_(1, 27)_ = 4.15, *p* = 0.042; GluA4, *H*_(1, 27)_ = 0.34, *p* = 0.56; GluK2, *H*_(1, 28)_ = 6.77, *p* = 0.0092; GluK3, *H*_(1, 27)_ = 5.65, *p* = 0.017; GluK4, *H*_(1, 28)_ = 3.14, *p* = 0.076; GluK5, *H*_(1, 27)_ = 8.29, *p* = 0.0040; GluN1, *H*_(1, 28)_ = 11.46, *p* = 0.0007; GluN2A, *H*_(1, 28)_ = 4.59, *p* = 0.032; GluN2B, *H*_(1, 27)_ = 3.58, *p* = 0.058; GluN2D, *H*_(1, 27)_ = 5.95, *p* = 0.015. ^*^p < 0.05; ^**^p < 0.01; ^***^p < 0.001.

Normalized average expression levels of the different glutamate receptor subunits in the HP-DG of individuals without alcohol dependence is shown in Figure 1. Qualitative estimation of high and medium expression was defined as equal to or greater than that of GluN1 and GluN2B, respectively. The results show a high expression of GluA2 and GluN1, medium expression of GluA1, GluA3, and GluN2B but lower expression of other ionotropic glutamate receptor subunit mRNAs. We then examined if the expression levels varied between controls and alcoholics. The levels of mRNAs encoding glutamate receptor subunits GluA2, GluA3, GluK2, GluK3, GluK5, GluN1, GluN2A, GluN2C, GluN2D, and GluN3A were significantly higher in the HP-DG of alcoholics as compared to controls (Figure 2). The mRNA levels of the affected subunits were increased about 50% or more in the HP-DG of the alcoholics as compared to the controls (Table 1). Remarkably, the mRNA encoding the obligatory NMDA receptor GluN1 subunit had almost doubled in abundance. Age, brain pH, PMI, smoking history, or RQI did not affect the significance between the two groups. No individual in either group consistently expressed genes at a higher level than the other individuals in the same group. For all the brain regions examined in this study, box and whiskers plots with median and whiskers were plotted using the Tukey method to determine outliers (data points above or below the whiskers). Statistical analysis was then performed where the outliers were not included when the data sets were compared. The subunit mRNA levels for the remaining subunits were not altered between the two groups in the HP-DG brain region (Figure 2).

**Table 1:** **Fold increase in expression of ionotropic glutamate receptor subunits in alcoholics compared to non-alcoholic individuals**.

	**HP-DG**	**OFC**
GluA2	1.67	
GluA3	1.69	
GluK2	1.84	
GluK3	2.66	
GluK5	1.50	
GluN1	1.90	
GluN2A	1.49	
GluN2C	1.50	
GluN2D	1.73	
GluN3A	1.65	1.42

*HP-DG, hippocampal dentate gyrus; OFC, orbitofrontal cortex Fold increase is calculated by: Average 2^−ΔCt^ of alcoholic samples/Average 2^−ΔCt^ of control samples*.

### Increased expression of GluN3A NMDA receptor subunit mRNA in the orbitofrontal cortex of alcoholics

**Figure 3:**
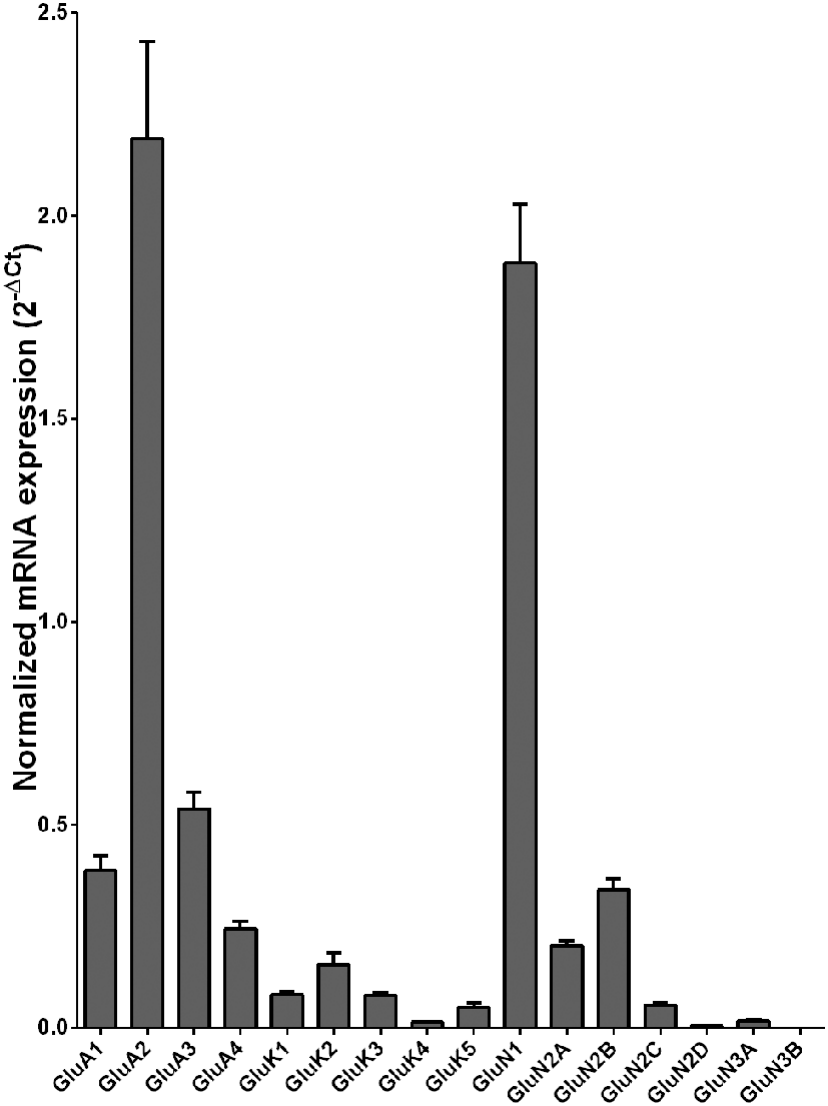
**Expression of ionotropic glutamate receptor subunit mRNAs in the orbitofrontal cortex of controls (*n* = 14)**. The mRNA level of each subunit was normalized to reference genes *PPIA* and *PGK1* and presented as mean ± SEM.

**Figure 4:**
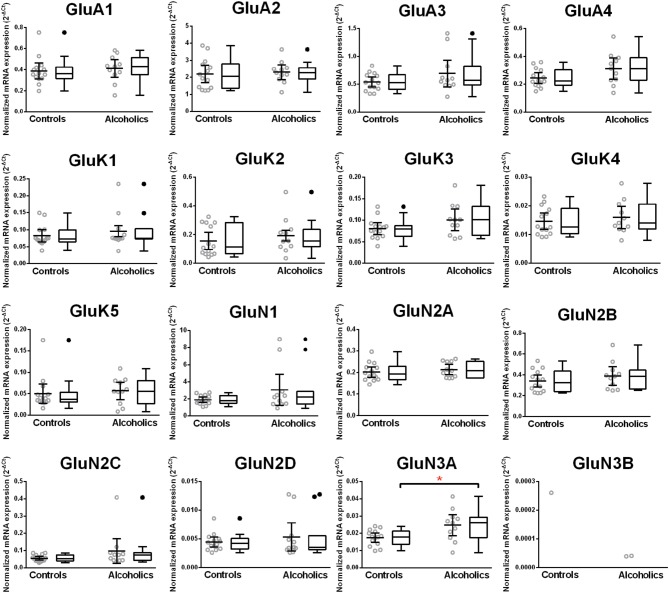
**Expression of ionotropic glutamate receptor subunits mRNA in the orbitofrontal cortex of controls (*n* = 15) and alcoholics (*n* = 14)**. Data from each group were presented as scatter dot plot (°) with mean and 95% confidence interval and box and whiskers plot with median and whiskers plotted by Tukey method to determine outliers (•-above or below the whiskers). Statistical analysis was performed after excluding outliers. One Way ANOVA with Bonferroni *post-hoc* test, GluA1, *df* = 20, *p* = 0.31; GluA2, *df* = 20, *p* = 0.71; GluA4, *df* = 20, *p* = 0.25; GluK4, *df* = 20, *p* = 0.99; GluK5, *df* = 20, *p* = 0.15; GluN1, *df* = 20, *p* = 0.96; GluN2A, *df* = 20, *p* = 0.56; GluN2B, *df* = 20, *p* = 0.23; GluN2D, *df* = 20, *p* = 0.38; GluN3A, *df* = 20, *p* = 0.017. Kruskal–Wallis ANOVA on ranks with Dunn's *post-hoc* test, GluA3, *H*_(1, 24)_ = 0.28, *p* = 0.60; GluK1, *H*_(1, 23)_ = 0.10, *p* = 0.75; GluK2, *H*_(1, 24)_ = 0.28, *p* = 0.60; GluK3, *H*_(1, 24)_ = 3.55, *p* = 0.06; GluN2C, *H*_(1, 24)_ = 0.99, *p* = 0.32. ^*^p < 0.05.

Normalized average expression levels of the different glutamate receptor subunits in the OFC of individuals without alcohol dependence is shown in Figure 3. Again, high and medium expression was defined as equal to or greater than that of GluN1 and GluN2B, respectively. In the OFC the results show a high expression of GluA2 and GluN1, medium expression of GluA1, GluA3, and GluN2B but lower expression of other glutamate receptor subunit mRNAs (Figure 3). We then examined if the expression levels varied between controls and alcoholics. Interestingly, the only change in the mRNA levels was an increase in GluN3A mRNA in samples from alcoholics as compared to controls (Figure 4 and Table 1). GluN3B mRNA expression was only identified in three individuals and the average mRNA expression of other subunits did not differ between the two groups (Figure 4). Age, brain pH, PMI, smoking history, or RQI did not affect the significance between the two groups.

### Unaltered expression of glutamate receptor subunit mRNAs in the dorso-lateral prefrontal cortex of alcoholics

**Figure 5:**
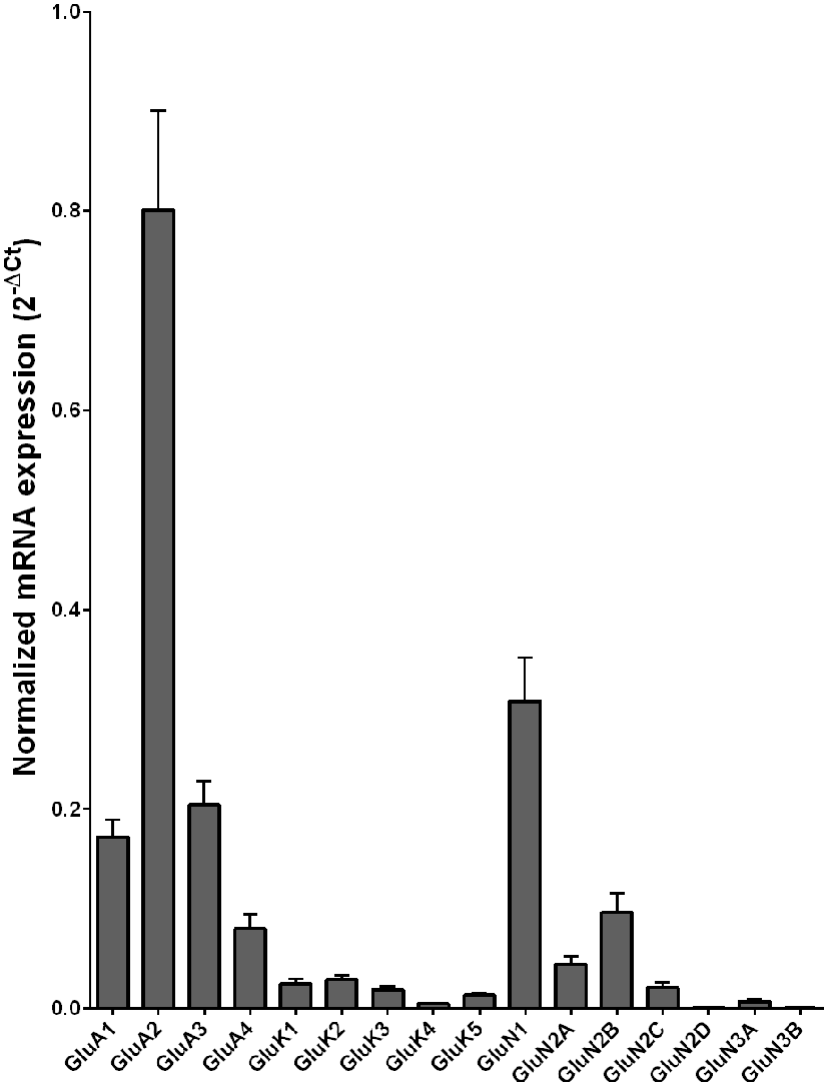
**Expression of ionotropic glutamate receptor subunit mRNAs in the prefrontal cortex of controls (*n* = 15)**. The mRNA level of each subunit was normalized to reference genes *ACTB* and *RPLP0* and presented as mean ± SEM.

**Figure 6:**
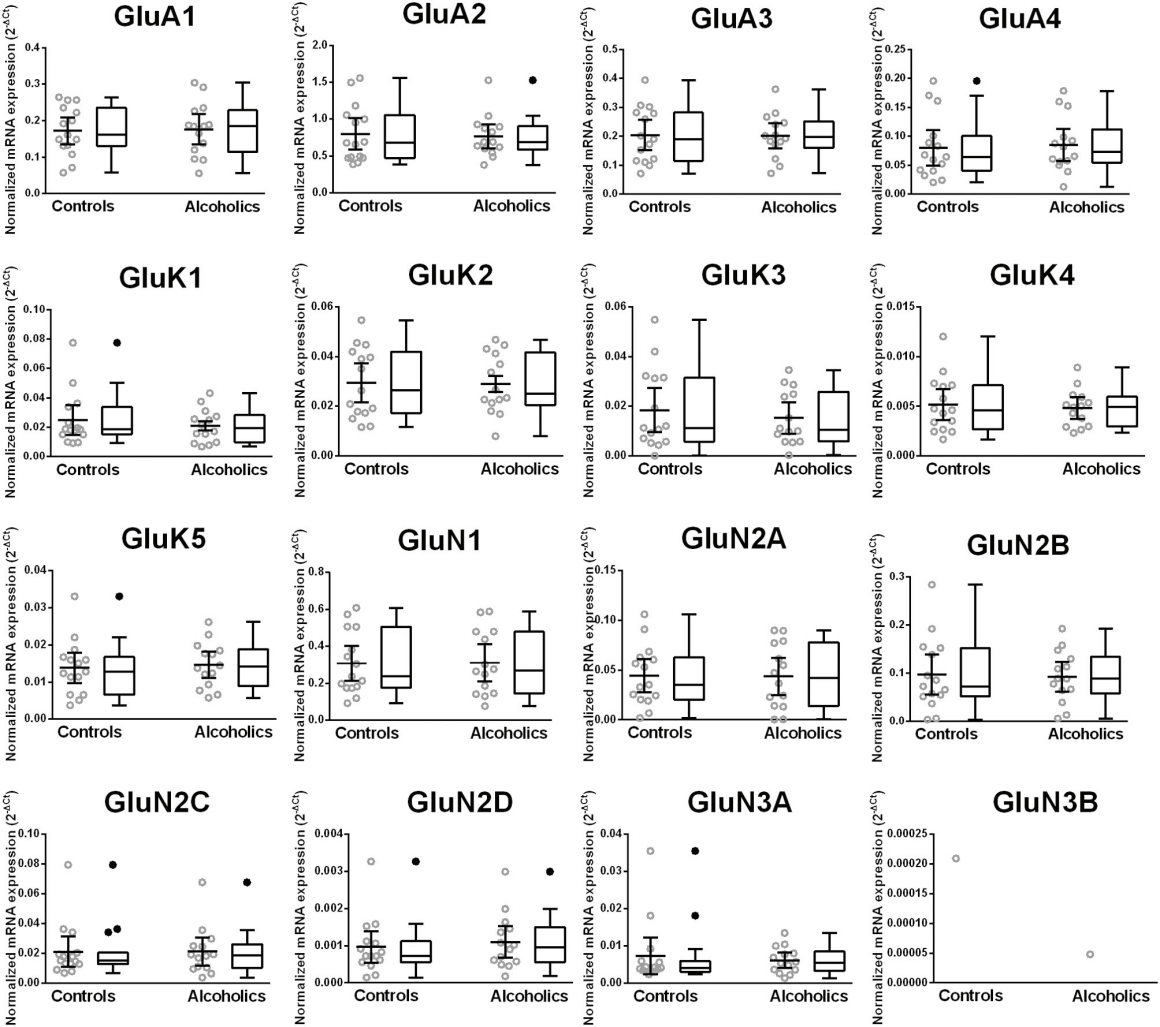
**Expression of ionotropic glutamate receptor subunits mRNA in the prefrontal cortex of controls (*n* = 14) and alcoholics (*n* = 11)**. Data from each group were presented as scatter dot plot (°) with mean and 95% confidence interval and box and whiskers plot with median and whiskers plotted by Tukey method to determine outliers (•-above or below the whiskers). Statistical analysis was performed after excluding outliers. One Way ANOVA with Bonferroni *post-hoc* test, GluA1, *df* = 23, *p* = 0.92; GluA3, *df* = 23, *p* = 0.91; GluK2, *df* = 23, *p* = 0.67; GluK4, *df* = 23, *p* = 0.52; GluK5, *df* = 23, *p* = 0.38; GluN2A, *df* = 23, *p* = 0.84; GluN2B, *df* = 23, *p* = 0.59; GluN2C, *df* = 23, *p* = 0.23; GluN2D, *df* = 23, *p* = 0.54. Kruskal–Wallis ANOVA on ranks with Dunn's *post-hoc* test, GluA2, *H*_(1, 28)_ = 0.26, *p* = 0.87; GluA4, *H*_(1, 28)_ = 0.54, *p* = 0.46; GluK1, *H*_(1, 28)_ = 0.0021, *p* = 0.96; GluK3, *H*_(1, 29)_ = 0.12, *p* = 0.73; GluN1, *H*_(1, 29)_ = 0.0019, *p* = 0.97; GluN3A, *H*_(1, 27)_ = 1.59, *p* = 0.21.

The mRNA expression profile of glutamate receptor subunits in the DL-PFC in individuals without alcohol dependence resembles that observed in the HP-DG and OFC (Figures 1, 3, and 5). Similar to the other two brain regions, high, and medium expression was defined as equal to or greater than that of GluN1 and GluN2B, respectively. There was a high expression of GluA2 and GluN1, medium expression of GluA1, GluA3, and GluN2B but lower expression of other glutamate receptor subunit mRNAs (Figure 5). GluN3B mRNA expression was detected only in two individuals. We then examined if expression levels varied between controls and alcoholics. No significant difference was detected in mRNAs expression levels for any subunit between the two groups (Figure 6). Age, brain pH, PMI, smoking history, or RQI did not affect the significance between the two groups.

## Discussion

In our present study on post-mortem brains of alcoholics, the effect of alcohol on the expression of specific glutamate receptor subunits mRNA varied widely between regions. The greatest difference was observed in the hippocampal dentate gyrus, where the expression levels of ten different subunits were increased in alcoholics, whereas in the orbitofrontal cortex only one was increased and in the prefrontal cortex none, as compared to controls. Gene expression profiling studies like RT-qPCR that use human autopsy brain tissue can be affected by many factors, and, therefore, all individuals included in the study were Caucasian males and the two groups were matched for age and PMI. In agreement with other studies on human post-mortem brain samples (Mayfield et al., 2002; Flatscher-Bader et al., 2005, 2006; Kalsi et al., 2009; Jin et al., 2011; Ponomarev et al., 2012), our study further demonstrated that expression changes were brain-region specific, suggesting that distinct functional domains in the brain may be differentially regulated and affected by ethanol.

The glutamate receptors are tetrameric cation-selective ion channels that are gated by glutamate. Functional glutamate receptors are formed exclusively by subunits within the same family and the subunit composition primarily determines the biophysical and the pharmacological properties of the receptors (Smart and Paoletti, 2012). However, glutamate receptors in the brain are known to exist in complexes with auxillary proteins that regulate the ion channel function and some of these proteins may be required for ethanol effects on neurotransmission (Moykkynen and Korpi, 2012; Yan and Tomita, 2012). In general, ethanol inhibits function of all ionotropic glutamate receptor classes but experimental conditions seem to produce a lot of variability in the results (Gass and Olive, 2008; Moykkynen and Korpi, 2012). NMDA receptors are regarded as the most sensitive to ethanol being inhibited by clinically relevant concentrations of ethanol (20 mM). The mechanism of inhibition of glutamate receptors by ethanol is nevertheless poorly understood and identifying the binding site has been difficult and hindered by the low-affinity nature of the ethanol binding at the receptors. For the NMDA receptors, results from point mutation studies of heterologously expressed receptors indicate the trans-membrane regions are important for the ethanol sensitivity (Smothers and Woodward, 2006; Xu et al., 2012). For the AMPA receptors, ethanol inhibition appears to be mediated by enhanced desensitization of the receptors induced by ethanol (Moykkynen et al., 2003).

How the ethanol's inhibition of the glutamate receptors then translates to altered expression of the receptors is not understood today. Chronic ethanol treatment increases the number of NMDA receptors in the brain (Moykkynen and Korpi, 2012), while less is known about the AMPA and kainate receptors. The GluN1 subunit has several splice variants, and chronic ethanol treatment differently affects some of these variants in brain region-specific manner (Raeder et al., 2008). In the present study we have not addressed the potential differential effects on splice variants of the receptor genes. Furthermore, effects of drugs non-competitively antagonizing NMDA receptors, such as dizocilpine and phencyclidine, can be partially generalized to the actions of ethanol (Bowen et al., 1997; Hundt et al., 1998) and they can also reduce voluntary alcohol drinking and alcohol deprivation effect reflecting relapse (Holter et al., 1996; Hundt et al., 1998; Vengeliene et al., 2005). A number of genetic mouse models of other ionotropic glutamate receptors have been tested for ethanol effects. GluA1 knockout mice do not show any alterations in ethanol dependence as compared to wild type animals (Cowen et al., 2003), whereas GluA3 knockout mice display deficient alcohol deprivation effect and relapse (Sanchis-Segura et al., 2006). These results suggest that glutamate neuroplasticity, which often is dependent on synaptic targeting of the AMPA receptor GluA1 and GluA3 subunits, is important in several processes of alcoholism. Alcohol affects the transcription, translation and levels of glutamate receptor subtypes in the brain by influencing a multitude of mechanisms (Chandrasekar, 2013), but most interestingly, our results suggest a very strict brain regional specificity for the effects of alcohol on the expression of ionotropic glutamate receptors in the human brain.

In the brain samples examined in this study a number of changes were identified in the hippocampal dentate gyrus region for the ionotropic glutamate receptors; two AMPA (GluA2, GluA3), three kainate (GluK2, GluK3, and GluK5), and five NMDA (GluN1, GluN2A, GluN2C, GluN2D, and GluN3A) receptor subunits mRNAs were increased in the brains of alcoholics. These results suggest that cellular events involving glutamate signaling in the brains of alcoholics e.g., synaptic strength (AMPA receptors), presynaptic release (kainate receptors) and neuronal plasticity (NMDA receptors) were probably changed in targeted neuronal populations of the hippocampus as a result of alcoholism. The changes in mRNA levels do not directly address whether protein levels of glutamate receptors have changed but this could potentially be verified by Western blot analysis of protein expression. The data complement previous studies on the effects of alcohol on glutamate signaling (Wernicke et al., 2003; Petrakis et al., 2004; Rujescu et al., 2005; Kim et al., 2006; Preuss et al., 2006; Kranzler et al., 2009; Ray et al., 2009; Ridge et al., 2009) and provide further evidence for long-term changes of glutamate receptors in the central nervous system by chronic alcohol consumption. The hippocampus is important for learning and memory (Nicoll and Roche, 2013). Processes like long-term potentiation (LTP) and long-term depression (LTD) are the experimental manifestations of neuronal plasticity. Long-term plasticity is known to involve glutamate receptors for induction and expression (Nicoll and Roche, 2013). Alterations in these processes contribute to the decrease in cognitive performance and memory impairment related to heavy ethanol intoxication (Heffernan, 2008; Loeber et al., 2009; Moykkynen and Korpi, 2012).

Compelling epidemiological evidence indicates that more than 50% of the risk for becoming an alcoholic stems from genetic susceptibility (Kalsi et al., 2009). GluN1, GluN2A, GluN2B, GluN2C, and GluK3 have all been identified as risk genes for alcoholism and alcohol-related phenotypes in human studies (Wernicke et al., 2003; Petrakis et al., 2004; Rujescu et al., 2005; Kim et al., 2006; Preuss et al., 2006; Kranzler et al., 2009; Ray et al., 2009; Ridge et al., 2009). In non-human primates, alternative splicing of GluA3 and GluA4 AMPA receptor subunits are regulated by ethanol consumption (Acosta et al., 2011). We have, in addition, identified in humans that the expression of GluA2, GluA3, GluK2, GluK5, GluN2D, and GluN3A genes change in the hippocampal DG and that of GluN3A in the OFC in response to chronic alcohol consumption. The extensive changes in the hippocampus can be expected to remodel neurotransmission and adapt the neuronal networks to long-term alcohol exposure (Gass and Olive, 2008). When neurons adjust their intrinsic excitability the fine balance between excitation and inhibition is altered and may destabilize network excitability (Yizhar et al., 2011; Remme and Wadman, 2012). We have previously shown, in the same post-mortem samples as used in this study, that specific GABA-A receptor subunits are up-regulated (Jin et al., 2011). Together the results from our studies on these samples indicate that the balance between excitation and inhibition may potentially be maintained by adjusting the expression levels of the neurotransmitter receptors involved. Similarly, in the DL-PFC of cynomolgus monkeys, the expression levels of glutamate and GABA-A receptor mRNAs are altered in the same direction upon alcohol exposure (Hemby et al., 2006; Acosta et al., 2011). It is possible that the ethanol inhibition of ionotropic glutamate receptors decreases neuronal excitability, which leads to compensatory up-regulation of the hippocampal glutamate receptor mRNAs. But, whether the change in one receptor system drives the change in the other cannot be deduced from our studies. In order to maintain a stable, adapting neuronal network with all neurons functioning within their dynamic range, a certain balance between excitation and inhibition may be required (Yizhar et al., 2011; Remme and Wadman, 2012). Indeed, when GABA-A receptors were ectopically expressed in mouse hippocampal neurons, adjustments in the glutamate receptors were observed (Moykkynen et al., 2007).

In conclusion, the results demonstrate that the excessive long-term alcohol consumption is associated with altered expression of genes encoding ionotropic glutamate receptors in a brain region-specific manner. It is an intriguing possibility that genetic predisposition to alcoholism may contribute to these gene expression changes.

## Author contributions

Igor Bazov, Olga Kononenko, Georgy Bakalkin obtained the material and made the mRNA from the tissue, Zhe Jin and Amol K. Bhandage designed primers and ran the qPCR, Zhe Jin, Esa R. Korpi and Bryndis Birnir designed the experiments, Amol K. Bhandage and Zhe Jin made the figures and did the statistical analysis, Bryndis Birnir wrote the paper that was edited by Esa R. Korpi and Zhe Jin and then commented on by other authors.

### Conflict of interest statement

The authors declare that the research was conducted in the absence of any commercial or financial relationships that could be construed as a potential conflict of interest.
