# Supplementary material for: Selective increases of AMPA, NMDA, and kainate receptor subunit mRNAs in the hippocampus and orbitofrontal cortex but not in prefrontal cortex of human alcoholics
Source: Front Cell Neurosci. 2014 Jan 29;8:11. doi: 10.3389/fncel.2014.00011 (PMC3905203; doi:10.3389/fncel.2014.00011)
Supplement: Supplementary file 1 [file DataSheet1.PDF]

Table S1. Demographic data of controls and alcoholics.

Subject No.	Age (Years)	PMI (Hours)	Brain pH	Smoking history	Brain regions	Cause of death	Toxicology at death
**CONTROLS**							
**1**	34	20.5	6.73	Yes	DL-PFC, OFC	Acute exacerbation of asthma	None
**2**	78	6.5	6.2	No	DL-PFC, OFC	Adenocarcinoma	NA
**3**	63	72	6.9	Yes	DL-PFC, OFC, HP-DG	Coronary artery atherosclerosis	NA
**4**	82	23.5	6.4	NA	DL-PFC, OFC	Sepsis	NA
**5**	38	13.5	6.26	Yes	DL-PFC, OFC, HP-DG	Atherosclerotic cardiovascular disease	NA
**6**	69	16	6.6	Yes	DL-PFC, OFC, HP-DG	Atherosclerotic cardiovascular disease	None
**7**		24	6.53	Yes	DL-PFC, OFC, HP-DG	Coronary artery atheroma	NA
**8**	59	20	6.56	Yes	DL-PFC, OFC, HP-DG	Coronary thrombosis	NA
**9**	56	25	6.1	No	DL-PFC	Ischemic heart disease	Codeine, morphine, naproxen
**10**	56	37	6.76	Yes	DL-PFC, OFC, HP-DG	Left ventricular scarring, hypertension, cardiomegaly	NA
**11**	82	36	6.24	No	DL-PFC, OFC, HP-DG	Myocardial infarction	NA
**12**	44	50	6.6	Yes	DL-PFC, OFC	Ischemic heart disease	NA
**13**	66	22	6.31	NA	HP-DG	Respiratory failure	NA
**14**	56	48	6.49	Yes	HP-DG	Cardiac failure	NA
**15**	43	66	6.2	No	HP-DG	Aspiration pneumonia	None
**16**	58	12	6.46	Yes	HP-DG	Ischemic heart disease	None
**17**	37	24	6.37	NA	HP-DG	Electrocution	None
**18**	46	25	6.65	NA	HP-DG	Mitral valve prolapse	None
**19**	58	15	6.71	No	HP-DG	Perforated gastric ulcer	NA
**20**	68	22	6.59	No	HP-DG	Asphyxia	Citralopram, Thioridazine, Mianserin
**21**	53	16	6.84	No	DL-PFC, OFC	Dilated cardiomyopathy	Lignocaine

**ALCOHOLICS**							
**1**	70	62	6.82	Yes	HP-DG	Cardiomyopathy	None
**2**	38	22	6.78	Yes	HP-DG	Ischemic heart disease	NA
**3**	34	8.5	6.61	Yes	DL-PFC, OFC, HP-DG	Hanging	Blood alcohol
**4**	77	20	6.34	Yes	DL-PFC, OFC, HP-DG	Bronchopneumonia	NA
**5**	65	32	5.66	NA	DL-PFC	Complications of chronic alcoholism	NA
**6**	50	17	6.3	No	HP-DG	Ischemic heart disease	None
**7**	79	48	6.34	Yes	DL-PFC, OFC, HP-DG	Ischemic heart disease	NA
**8**	39	24	6.56	Yes	DL-PFC, OFC, HP-DG	Aortic stenosis	NA
**9**	56	22	6.52	Yes	DL-PFC, OFC, HP-DG	Gastro-intestinal hemorrhage	NA
**10**	59	24	6.57	No	DL-PFC, OFC	Cardiomyopathy	NA
**11**	56	15	6.66	NA	DL-PFC, OFC, HP-DG	Ischemic heart disease and emphysema	Nordiazepam
**12**	56	45	6.51	NA	DL-PFC, OFC, HP-DG	Bleeding esophageal varices	Blood alcohol
**13**	44	15	6.48	No	DL-PFC, OFC, HP-DG	Ischemic heart disease	Diazepam, noediazepam
**14**	81	36	6.44	Yes	DL-PFC, OFC, HP-DG	Sepsis	NA
**15**	62	49	6.49	Yes	DL-PFC	Ischemic heart disease	Sertraline
**16**	66	11.5	6.4	Yes	DL-PFC	Pneumonia	None
**17**	53	57	6.75	Yes	DL-PFC, OFC, HP-DG	Chronic airflow limitation	NA
**18**	61	24	6.52	Yes	DL-PFC, OFC	Ischemic heart disease	NA
**19**	57	18	6.6	Yes	DL-PFC, OFC	Ischemic heart disease	NA

PMI, post-mortem interval; DL-PFC, dorsolateral prefrontal cortex; OFC, orbitofrontal cortex; HP-DG, hippocampal dendate gyrus; NA, not available.

Table S2. Primers list for RT-qPCR.

Genes	Accession no.	FD Primer Sequence	RV Primer Sequence	Amplicon Size (bp)
GRIA1 (GluA1)	NM_000827	ACTGGAAGAGACCCAAGTACACCTC	AGACAATCCCCAGCATTCCCCC	127
GRIA2 (GluA2)	NM_000826	CGCAGTCACTAATGCTTTCTGCTCC	AGGAGACGTGGAGTGTTCCGCA	119
GRIA3 (GluA3)	NM_000828	ACACCATCAGCATAGGTGGA	CTTCTCGGTGGTGTTCTGGT	107
GRIA4 (Glu42)	NM_000829	GGACACTCAAACAGGTTCG	TTGTGTAATTGACTCTACGTCC	80
GRIK1 (GluK1)	NM_000830	AGCAACAAAGACAAGTCCA	GGGTTCTTCCAGAATGGTG	81
GRIK2 (GluK2)	NM_021956	AAAGTGGTCGATGGAACGA	TAGAGCAGCATCAGTCGTC	85
GRIK3 (GluK3)	NM_000831	AGAGAGCAGCGTTCCTTCTG	CGGCGGTCATTGAA TGTGT	146
GRIK4 (GluK4)	NM_014619	TGAGGATCGCTGCTATCTTGG	CGTACTCGCTGTCTCTGAGAA	159
GRIK5 (GluK5)	NM_002088	CAGGTGCTCTCATCACTGCG	CTGACACATGGTGTCCGTGGT	198
GRIN1 (GluN1)	NM_000832	CATCCTCAAGTCCCACGAG	TTCCTGATACCGAACCCAC	67
GRIN2A (GluN2A)	NM_000833	GAATGATCGGTGAAGTGGTC	CCACTTCAGAACGTTCCTC	81
GRIN2B (GluN2B)	NM_000834	GGCAGATAAGGATGAATCCTC	ATGATGTTGAGCATTACGGA	81
GRIN2C (GluN2C)	NM_000835	CTTCTTGGAGCCATATAGCC	AACATGAAGACGGTGATGG	85
GRIN2D (GluN2D)	NM_000836	ATGGTTTCCTTCCTGAGCT	ATGAAGTACCTATGCAGACTCTC	82
GRIN3A (GluN3A)	NM_133445	GCCATAGAAGGATACGGCA	CTTGTATTGACTGATTAGCTCGG	81
GRIN3B (GluN3B)	NM_138690	CCTGTCCGAGTTCATCAG	CTGCAGGGTCTCTGTAAC	118
ACTB (β-actin)	NM_001101.2	CCT GGC ACC CAG CAC AAT	GGG CCG GAC TCG TCA TAC T	144
RPLP0	NM_001002	CCTCATATCCGGGGGAATGTG	GCAGCAGCTGGCACCTTATTG	95
UBC	NM_021009	CGGTGAACGCCGATGATTAT	ATCTGCATTGTCAAGTGACGA	124
PPIA	NM_021130	CCCACCGTGTTCTTCGACAT	CCAGTGCTCAGAGCACGAAA	116
PKG1	NM_000291	AGGGAAAAGATGCTTCTGGG	AAGTGAAGCTCGGAAAGCTTCTAT	71

Table S3. Analysis of normality of RT-qPCR data distribution by Shapiro–Wilk normality test.

	**HP-DG**	**OFC**	**DL-PFC**
GluA1	< 0.05	= 0.9	= 0.76
GluA2	< 0.05	= 0.25	< 0.05
GluA3	< 0.05	< 0.05	= 0.46
GluA4	< 0.05	= 0.13	< 0.05
GluK1	= 0.56	< 0.05	< 0.05
GluK2	< 0.05	< 0.05	= 0.09
GluK3	< 0.05	< 0.05	< 0.05
GluK4	< 0.05	= 0.11	= 0.06
GluK5	< 0.05	= 0.41	= 0.73
GluN1	< 0.05	= 0.43	< 0.05
GluN2A	< 0.05	= 0.2	= 0.18
GluN2B	< 0.05	= 0.058	= 0.11
GluN2C	= 0.75	< 0.05	= 0.38
GluN2D	< 0.05	= 0.28	= 0.31
GluN3A	= 0.098	= 0.4	< 0.05

$p<0.05$ indicates the data are not normally distributed. HP-DG, hippocampal dendate gyrus; OFC, orbitofrontal cortex; DL-PFC, dorsolateral prefrontal cortex.

Table S4. Sample demographic information.

Characteristics	Hippocampal dentate gyrus			Dorsolateral prefrontal cortex			Orbitofrontral cortex		
	**Controls**	**Alcoholics**	***p* Value**	**Controls**	**Alcoholics**	***p* Value**	**Controls**	**Alcoholics**	***p* Value**
Number	15	13		15	14		14	11	
Age (years)	57±3	56±4	0.909	59±4	59±4	0.981	59±4	58±5	0.774
PMI (h)	30±4.8	30±4.9	0.908	27±4.2	29±4.2	0.777	27±4.6	29±4.7	0.763
Brain pH	6.5±0.05	6.5±0.05	0.599	6.5±0.06	6.5±0.07	0.458	6.6±0.06	6.5±0.13	0.714
RNA quality indicator	6.9±0.23	6.6±0.14	0.23	7.3±0.31	7.8±0.31	0.206	7.7±0.99	7.6±0.96	0.936
Smoking history*	8(67%) S, 4(33%) NS	9(82%) S, 2(12%) NS	0.64	10(77%)S, 3(23%) NS	9(82%)S, 2(18%)NS	1.0	10(77%)S, 3(23%)NS	7(78%)S, 2(22%) NS	1.0

PMI, post-mortem interval; S,smoker; NS,non-smoker.

Age, PMI, brain pH, and RNA quality indicator are shown as mean±SE, and the difference between controls and alcoholics was tested with Student's t-test or Mann–Whitney U-test.

*Smoking histories are not available for all subjects. The proportion of smokers and non-smokers between controls and alcoholics was tested with Fisher's exact test.
